# Preventing Suicide

**Published:** 1873-09

**Authors:** 


### PREVENTING SUICIDE.

Pass a law that every suicide shall be boiled until all the flesh leaves the bones, and the skeleton hung up in the nearest doctor's shop, or at the first cross-roads.

More than a hundred years ago suicide became epidemic among women. When there were eleven cases within a few days the town authorities ordered that every body found should be hung up in its nude state to a tree at the entrance to the town. Not a single other case occurred from that day. Almost any crime may be prevented by making the punishment certain, prompt and disagreeable, or ridiculous, without its being cruel. Intemperance could be stampeded in this way a thousand years earlier than prohibitory laws will do it, although local prohibition is the best available remedy known as yet. We respectfully suggest a law to this effect: That any man or woman found the "worse for liquor" should be dressed in a pair of high topped boots, a night cap, and a single long white gown, then blindfolded; place a stake within twenty feet of a river bank, or large, half filled cistern; he or she to take a station twenty yards distant, with a wheelbarrow, with directions to "strike the stake," the reward for so doing to be "let off" for that time, or in default to keep on trying until at least three "duckings" shall "transpire," all the inhabitants round about being admitted to the show at one cent each. We have not taken out a patent for this "cure for drunkenness," but there is reason to believe that it would be marvellously successful, at least as an innocent amusement to all the little boys and girls.

